# The miR-17-92 Cluster and Its Target *THBS1* Are Differentially Expressed in Angiosarcomas Dependent on *MYC* Amplification

**DOI:** 10.1002/gcc.21943

**Published:** 2012-03-02

**Authors:** Antoine Italiano, Rachael Thomas, Matthew Breen, Lei Zhang, Aimee M Crago, Samuel Singer, Raya Khanin, Robert G Maki, Aleksandra Mihailovic, Markus Hafner, Tom Tuschl, Cristina R Antonescu

**Affiliations:** 1 Department of Pathology, Memorial Sloan-Kettering Cancer CenterNew York, NY; 2 Department of Medical Oncology, Institute BergoniéBordeaux, France; 3 Department of Molecular Biomedical Sciences, College of Veterinary MedicineNorth Carolina State University, Raleigh, NC; 4 Center for Comparative Medicine and Translational Research, North Carolina State UniversityRaleigh, NC; 5 Cancer Genetics Program, UNC Lineberger Comprehensive Cancer CenterChapel Hill, NC; 6 Department of Surgery, Memorial Sloan-Kettering Cancer CenterNew York, NY; 7 Computational Biology Center, Memorial Sloan-Kettering Cancer CenterNew York, NY; 8 Department of Medicine/Pediatrics, Mount Sinai School of MedicineNew York, NY; 9 Laboratory for RNA Molecular Biology, Howard Hughes Medical Institute, The Rockefeller UniversityNew York, NY

## Abstract

Angiosarcomas (ASs) represent a heterogeneous group of malignant vascular tumors that may occur spontaneously as primary tumors or secondarily after radiation therapy or in the context of chronic lymphedema. Most secondary ASs have been associated with *MYC* oncogene amplification, whereas the role of *MYC* abnormalities in primary AS is not well defined. Twenty-two primary and secondary ASs were analyzed by array-comparative genomic hybridization (aCGH) and by deep sequencing of small RNA libraries. By aCGH and subsequently confirmed by fluorescence in situ hybridization, MYC amplification was identified in three out of six primary tumors and in 8 out of 12 secondary AS. We have also found *MAML1* as a new potential oncogene in *MYC*-amplified AS. Significant upregulation of the miR-17-92 cluster was observed in *MYC*-amplified AS compared to AS lacking *MYC* amplification and the control group (other vascular tumors, nonvascular sarcomas). Moreover, *MYC*-amplified ASs were associated with a significantly lower expression of thrombospondin-1 (*THBS1)* than AS without *MYC* amplification or controls. Altogether, our study implicates *MYC* amplification not only in the pathogenesis of secondary AS but also in a subset of primary AS. Thus, MYC amplification may play a crucial role in the angiogenic phenotype of AS through upregulation of the miR-17-92 cluster, which subsequently downregulates *THBS1*, a potent endogenous inhibitor of angiogenesis. © 2012 Wiley Periodicals, Inc.

## INTRODUCTION

Angiosarcoma (AS) represents a rare (<2%) subgroup of soft tissue sarcomas characterized by an aggressive clinical behavior (Fletcher et al., 2002). Radical surgery and adjuvant radiotherapy, when indicated, represent the cornerstone of the treatment for patients with localized disease. However, despite an adequate locoregional treatment, up to 50% of patients will develop metastatic relapse and will die of disease (Fayette et al., 2007). The genetic and molecular aberrations involved in AS tumorigenesis remain poorly understood. We and others have recently shown that a particular subset of AS, those arising in a previously radiated area, is characterized by a consistent amplification of the *MYC* oncogene in chromosome band 8q24.21 (Manner et al., 2010; Guo et al., 2011). So far, such amplifications of *MYC* have not been reported in primary AS. *MYC* plays a crucial role in growth control, differentiation, and apoptosis, and its aberrant expression is associated with several cancers (Adhikary and Eilers, 2005; Albihn et al., 2010). Interestingly, recent studies have also demonstrated a major contribution of *MYC* to tumor angiogenesis (Baudino et al., 2002; Dews et al., 2006; Gordan et al., 2007; Dang et al., 2008). One of the major mechanisms involved in MYC-induced angiogenesis is the upregulation of the miR-17-92 microRNA cluster, the predicted targets of which include thrombospondin-1 (*THBS1*), encoding a potent endogenous inhibitor of angiogenesis, and connective tissue growth factor (*CTGF*), encoding an extracellular matrix-associated molecule involved in angiogenesis and metastatic progression (Dews et al., 2006). We hypothesize that this molecular process is frequent in AS, at least in those tumors carrying an amplification of *MYC*, and may play a crucial role in the tumorigenesis of such vascular tumors. Thus, we used genome-wide array comparative genomic hybridization and deep sequencing of small RNA libraries in a group of 22 AS patients to investigate this pathogenetic mechanism.

## MATERIALS AND METHODS

### Patient and Samples

Twenty-two AS and four other vascular tumors (two epithelioid hemangiomas and two epithelioid hemangioendotheliomas) were included in this study on the basis of availability of frozen material for molecular studies. The clinicopathologic characteristics are summarized in Table 1. Diagnoses were established according to the World Health Organization Classification of Tumors (Fletcher et al., 2002). This study was approved by the institutional review board.

**TABLE 1:** Clinicopathologic Characteristics of AS and Types of Platforms Investigated

Case ID	Age	Sex	Previous RT-therapy	Chronic Lymphedema	Primary location	MYC amplified by FISH	MYC amplified by aCGH	Micro-RNA deep sequencing	qRT-PCR
AS3^a^	58	M	No	No	Femur	NA	Yes	No	Yes
AS4	63	F	Yes	No	Breast	Yes	NA	Yes	No
AS9	70	M	No	No	Thigh	No	No	Yes	Yes
AS10	70	F	Yes	No	Breast	Yes	Yes	No	Yes
AS11	50	M	No	No	Spleen	NA	No	Yes	No
AS15	61	F	Yes	No	Breast	NA	Yes	No	Yes
AS20	56	F	Yes	No	Breast	Yes	Yes	No	Yes
AS27	37	F	No	No	Breast	No	NA	Yes	No
AS29	75	F	Yes	No	Breast	Yes	No	Yes	Yes
AS30	38	F	Yes	No	Head and neck	No	No	Yes	Yes
AS31	79	M	No	No	Scalp	No	NA	Yes	Yes
AS32	76	F	Yes	No	Breast	Yes	Yes	No	Yes
AS38	74	F	Yes	Yes	Forearm	Yes	Yes	Yes	Yes
AS39	84	M	Yes	Yes	Arm	Yes	Yes	Yes	Yes
AS68	80	F	Yes	No	Breast	Yes	Yes	Yes	No
AS70^a^	38	F	No	No	Breast	Yes	Yes	Yes	Yes
AS73	83	F	Yes	No	Breast	NA	Yes	Yes	No
AS76	76	M	Yes	No	Bladder	NA	No	Yes	No
AS85	76	F	Yes	No	Breast	Yes	NA	Yes	No
AS123	76	F	Yes	No	Breast	No	No	Yes	Yes
AS124	57	M	No	No	Head and neck	NA	No	Yes	No
AS125^a^	73	F	No	No	Breast	Yes	Yes	No	Yes

*Other vascular tumors*						

	Age	Sex	Histology	Primary location	Micro-RNA deep sequencing	qRT-PCR
AS78	M	68	EHE	Arm	No	Yes
AS80	M	41	Epithelioid hemangioma	Chest wall	Yes	No
AS84	M	31	Epithelioid hemangioma	Metatarsal bone	Yes	No
EHE74	M	44	EHE	Liver	No	Yes

RT, radiation therapy; EHE: epithelioid hemangioendothelioma.

a Primary AS tumors that showed MYC amplification by aCGH.

### Array-CGH

Genomic DNA was isolated from frozen tumor tissue by phenol/chloroform extraction and quality was confirmed by spectrophotometry and electrophoresis. Array CGH analysis was performed as described previously (Thomas et al., 2011). Briefly, tumor and reference DNA samples were labeled with Cyanine-3-dUTP and Cyanine-5-dUTP, respectively, by random priming (Agilent Enzymatic Labeling Kit, Agilent Technologies, Santa Clara, CA). The reference sample comprised a pool of DNA from multiple clinically healthy donors (Promega, Madison, WI) of the same gender as the AS patient. The labeled probes were combined and hybridized to a ∼180,000-feature, genome-wide oligonucleotide CGH array (design 052252, Agilent Technologies). Arrays were scanned at 5 μm resolution using an Agilent G2565CA scanner. Image data were processed with Feature Extraction version 10.10 and Genomic Workbench version 6.5 (Agilent Technologies). Data were filtered to exclude probes exhibiting nonuniform hybridization or signal saturation and were normalized using the centralization algorithm with a threshold of six. The ADM2 algorithm was used to define CNAs using a “three probes minimum” filter and a threshold of six with a fuzzy zero correction. A genomic copy number amplification was defined as a region within which the log_2_ ratio of tumor DNA to reference DNA exceeded 2.0. The distribution of aberrations within all tumors of the same subtype (primary or secondary) was evaluated using the “common aberration” function of Genomic Workbench, to identify statistically significant trends. The “differential aberration” function was similarly used to compare the genomic profiles of all primary tumors with all secondary tumors, to identify aberrations whose DNA copy number status was significantly associated with tumor subtype.

### Fluorescence In Situ Hybridization

Fluorescence in situ hybridization (FISH) analysis was performed by hybridization of bacterial artificial chromosome (BAC) probes, covering *MYC* (RP11-440N18; 8q24.21:128,596,756-128,777,986), *FLT4* (RP11-586L9; 5q35.3:179,971,355-180,139, 031), *MAML1* (RP11-828P1; 5q35.3:179,128,722-179,355,313) and two reference probes from the 5q33.3 region (RP11-583A20; chr5:158,433,114-158,602,540 and RP11-117N12; chr5:158,645,646-158,819,496) onto 4-μm sections of formalin-fixed paraffin-embedded tissue from each tumor. BAC clones were chosen according to their genomic location as defined in the UCSC genome browser (http://genome.ucsc.edu). The BAC clones were obtained from BACPAC sources of Children's Hospital of Oakland Research Institute (CHORI) (Oakland, CA) (http://bacpac.chori.org). BAC DNA was isolated according to the manufacturer's instructions, labeled with different fluorochromes in a nick translation reaction, denatured, and hybridized to pretreated slides. Slides were then incubated, washed, and mounted with DAPI in an antifade solution as described previously (Antonescu et al., 2010). The genomic location of each BAC set was verified by hybridizing them to normal metaphase chromosomes. Two hundred interphase nuclei from each tumor were examined using a Zeiss fluorescence microscope (Zeiss Axioplan, Oberkochen, Germany), controlled by Isis 5 software (Metasystems).

### Micro-RNA Sequencing

Total RNA was extracted from frozen tumor tissue using Trizol reagent according to the manufacturer's instructions (Invitrogen, Carlsbad, CA). Small RNA cDNA libraries were prepared from 16 AS and two other vascular tumors as described previously (Hafner et al., 2010). In 20-μl reactions, 2 μg total RNA was ligated to 100 pmol adenylated 3′ adapter containing a unique pentamer barcode at the 5′ end using 1 μg Rnl2(1-249)K227Q [purified from *Escherichia coli* containing pET16b-Rnl2(1-249)K227Q (Addgene, Cambridge, MA)], in 50 mM Tris-HCl, pH 7.6; 10 mM MgCl_2_; 10 mM 2-mercaptoethanol; 0.1 mg/mL acetylated bovine serum albumin (Sigma-Aldrich, St. Louis, MO) and 15% DMSO for 16 hr on ice. After ligation, up to 20 samples bearing unique barcodes were pooled and purified on a 15% denaturing polyacrylamide gel. RNAs of 45 and 50 nucleotides were excised from the gel, eluted, and ligated to 100 pmol 5′ oligoribonucleotide adapter (GUUCAGAGUUCUACAGUC CGACGAUC) as described above for the 3′ adaptors, except that reactions contained 0.2 mM ATP and RNL1 instead of RNL2(1–249) K227Q and were incubated for 1 hr at 37°C. Ligated small RNAs were purified on a 12% polyacrylamide gel, reverse transcribed using SuperScript III Reverse Transcriptase (Invitrogen, Carlsbad, CA), and amplified by PCR. The forward primer was AATGATACGGCGACCACCGACAGGTTCAGAGTTCTACAGTCCGA; reverse transcription and reverse primer was CAAGCAGAAGAC GGCATACGA. On average 1,265,133 (range, 332,816–2,543,130) sequence reads of miRNAs were obtained per sample.

### Real-time RT-PCR

One microgram of total RNA was reverse transcribed using the High-Capacity cDNA Reverse Transcription Kit (Applied Biosystems, Carlsbad, CA) at 25°C for 10 min, 37°C for 120 min, 85°C for 5 min and hold at 4°C. In all, 20 ng/μL of resultant cDNA was used in a Q-PCR reaction using an 7500 Real-Time PCR System (Applied Biosystems, Carlsbad, CA) and predesigned TaqMan ABI Gene expression Assays (Hs_ 01070499_m1 for *MAML1*; HS00905030_m1 for *MYC*; Hs_00962908_m1 for *THBS1*; HS_ 00170014_m1 for *CTGF*). Amplification was carried at 95°C for 10 min, and 40 cycles (95°C for 15 sec, 60°C for 1 min). To calculate the efficiency of the PCR reaction, and to assess the sensitivity of each assay, we also performed a 5-point standard curve (80, 26.67, 8.88, 2.96, and 0.98 ng/μL). Triplicates CT values were averaged, amounts of target will be interpolated from the standard curves and normalized to *GAPDH* (reference gene).

### Western Blotting

Western blotting was performed to assess the expression of MAML1 protein in AS with and without *MAML1* gene amplification. Frozen tissue from five AS samples (one with *MAML1* amplification and four without *MAML1* amplification) and cells from the *MYC*-amplified breast cancer cell line SKBR3 (Guo et al., 2011) were homogenized in RIPA buffer supplemented with protease and phosphatase inhibitors. Electrophoresis and immunoblotting were performed on the protein extracts using 50 μg of protein per sample and the anti-MAML1 monoclonal antibody (Cell Signaling Technology, Danvers, MA) was diluted according to the manufacturers' recommendations. Following hybridization with the secondary anti-rabbit antibody (Calbiochem, La Jolla, CA), the blots were incubated with Immun-Star horseradish peroxidase luminal/enhancer (Bio-Rad) and exposed onto Kodak Biomax MR Film (Eastman Kodak Co., Rochester, NY).

### Statistical Analysis

The count data were normalized/rescaled using DESeq R package (Anders and Huber, 2010). MicroRNAs with <10 counts in both type/condition were not considered for further analysis. To determine differentially expressed microRNAs a Binomial test implemented in DESeq, package was used with fold change of 2, and false discovery rate of 0.1.

## RESULTS

### *MYC* is Amplified in the Majority of Secondary AS but also in a Subset of Primary AS

**Figure 1:**
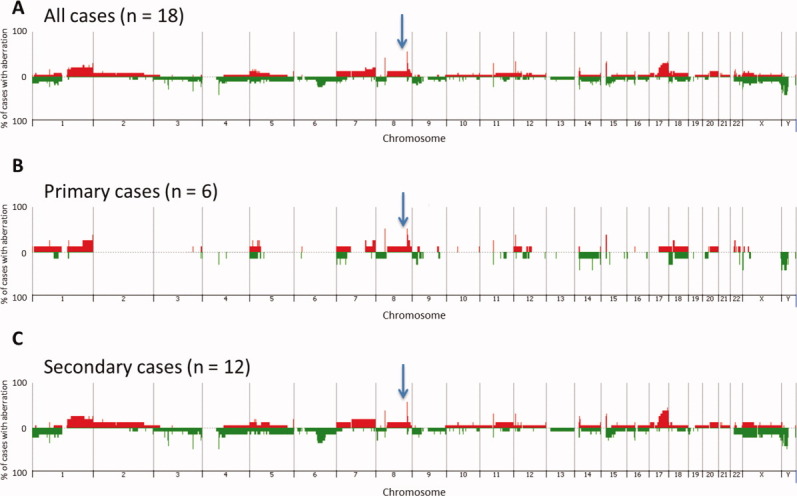
Penetrance plots showing the frequency of gain and loss of genomic regions within (A) all 18 AS evaluated by aCGH, (B) all primary tumors, and (C) all secondary tumors. Each chromosome is represented on the *x*-axis, and the *y*-axis indicates the % gain or loss of the corresponding genomic region within the corresponding population. Gains are shown in red and losses in green. The position of *MYC* genomic region is indicated by an arrow.

**Figure 2:**
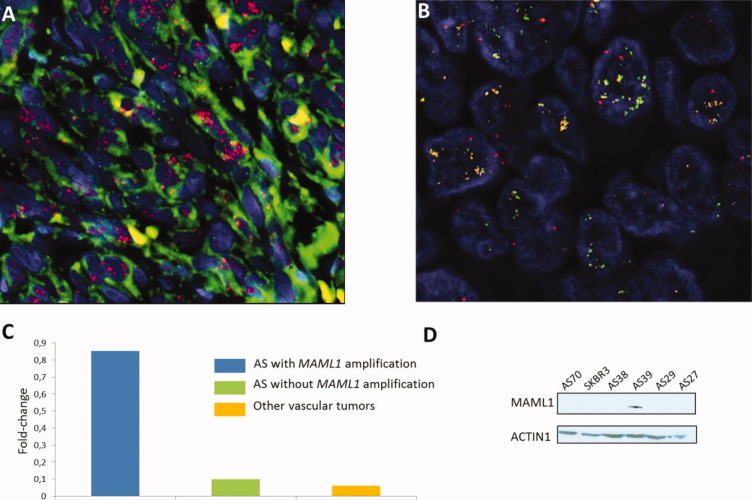
*MYC* and *MAML1* amplification in AS. (A) FISH analysis with BAC probes RP11-440N18 (*MYC*) and RP11-586L9 (*FLT4*), showing high level of *MYC* amplification (red signal) in a case of primary AS (AS125). *FLT4* (green signal) is not amplified. (B) FISH analysis with BAC probes RP11-828P1 (*MAML1*), RP11-586L9 (*FLT4*), and 5q33.3 region reference probes (RP11-583A20 and RP11-117N12; red) in a chronic lymphedema-associated AS (AS39). *MAML1* (orange signal) and *FLT4* (green signal) are coamplified. (C) QRT-PCR analysis measuring *MAML1* mRNA expression in AS with *MAML1* gene amplification, in AS without *MAML1* gene amplification and in other vascular tumors. Gene expression was quantified by QRT-PCR and expressed as mean relative expression (reference gene: *GAPDH*). (D) Western blotting assessing MAML1 protein expression in AS with *MAML1* gene amplification (AS 39), in AS without *MAML1* gene amplification (AS27, AS29, AS38, and AS70) and in the SKBR3 breast cancer cell line.

Eighteen cases of AS (6 primary AS and 12 secondary AS) were analyzed by array-CGH, each of which exhibited DNA copy number aberrations. The frequency and distribution of aberrations within the cohort is summarized as a penetrance plot in Figure 1, which also compares the profiles of genomic gains and losses in primary tumors with those of secondary tumors. A total of 438 aberrations was identified across the cohort of 18 cases, with a mean of 24 aberrations per case (median = 22). The incidence of aberrations within the 12 cases of secondary AS (total = 309 aberrations, range = 12–49, mean per case = 26, median = 23) was highly comparable to that of the six primary AS (total = 129 aberrations, range = 10–35, mean per case = 22, median = 20). No genomic regions showed statistically significant association with tumor subtype when evaluating all tumors within the same subtype, nor when comparing all primary tumors to all secondary tumors. Supporting Information Table 1 provides a summary of genomic imbalances within each of the 18 tumors evaluated by array-comparative genomic hybridization (aCGH). Strikingly, the *MYC* locus was found amplified in 8 out of the 12 secondary AS, but also in three out of the six primary AS. The amplification status of *MYC* was confirmed by FISH in all but two cases, showing consistently more than hundreds of copies in the form of hsr or multiple focal amplicons, compared to *FLT4* used as control (Fig. 2A). In one case, FISH results were not interpretable despite several attempts (AS3: primary AS arising from bone), whereas in the other case (AS29: radiation-induced breast AS), there was discordance between FISH showing a *MYC* amplification and array-CGH, showing no imbalance at this locus. Among the four secondary AS lacking *MYC* amplification, two of them were outside the breast, one in the parotid, and the other one in the bladder. In two of these cases, the lack of *MYC* abnormalities was validated by both aCGH and FISH, whereas in one case no material was available for FISH validation. *MYC* mRNA expression by real-time PCR was assessed in eight cases of AS [three primary AS with *MYC* amplification, three secondary AS with *MYC* amplification, and two AS without *MYC* amplification (used as control group)]. We found an overexpression of the *MYC* gene in secondary and primary AS with *MYC* amplification (mean fold changes = 2.5 and 2.4, respectively). There were no correlations found with AS histologic subtype (epithelioid, spindle), anaplasia or degree of vascular differentiation and the presence of *MYC* genomic aberrations.

### The NOTCH Pathway Effector Gene *MAML1* (5q35.3) is Amplified and Overexpressed in a Subset of AS

Two cases of *MYC*-amplified secondary AS displayed coamplification of the 5q35.3 region, which included *FLT4*, encoding the vascular endothelial growth factor receptor 3, as reported previously (Guo et al., 2011), as well as *MAML1,* which appears relevant for AS genesis because it encodes a crucial effector of the NOTCH pathway. As such, we further analyzed the amplification status of *MAML1* in 10 additional cases (two primary AS and eight secondary AS) by FISH. Overall, we have found an amplification of *MAML1* in 5 cases (all secondary AS) out of 28 samples (18%) (Fig. 2B). In all these cases, *MAML1* was coamplified with *FLT4*. To confirm that *MAML1* may be a “driver” gene of the 5q35.3 amplicon, we have assessed its mRNA by qRT-PCR (3 AS cases with *MAML1* amplification, 17 AS cases without *MAML1* amplification, and 2 other vascular tumors) and protein expression by Western blotting (one case with *MAML1* amplification, four AS cases without *MAML1* amplification and the SKBR3 cell line). By qRT-PCR, we have found a significant overexpression of *MAML1* in AS with *MAML1* amplification in comparison with AS without this aberration (*P* < 0.0001) and other vascular tumors (*P* < 0.0001) (Fig. 2C). By Western blotting, we observed that MAML1 protein was expressed in the AS cases with *MAML1* amplification, but not in the four AS cases lacking *MAML1* amplification nor in the *MYC*-amplified SKBR3 cell line (Fig. 2D). We did not observe any difference in terms of clinical or pathologic characteristics between AS with and without 5q35 amplification.

### The miR-17-92 Cluster is Preferentially Overexpressed in AS with *MYC* Amplification

**Figure 3:**
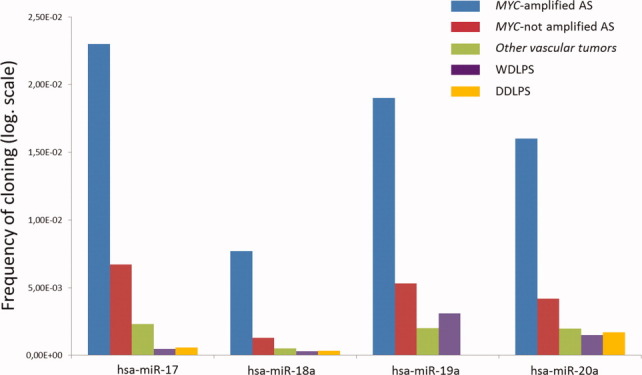
Differential expression of the miRNA of the miR-17-92 cluster in *MYC*-amplified AS, in *MYC*-unamplified AS, in other vascular tumors, in WDLPS and in DDLPS. (*y* axis: frequency of cloning: proportion of miRNA from the total, transformed by the log function).

We profiled miRNA expression in 16 AS (eight with *MYC* amplification and eight without *MYC* amplification) and two other vascular tumors using deep sequencing of small RNA libraries. By comparing the miRNA expression profile of *MYC*-amplified AS and *MYC* unamplified AS, we found 43 miRNAs that were differentially expressed (Table 2). Among them, miRNAs from the miR-17-92 cluster were the most strongly upregulated miRNAs (Fig. 3 and Table 2). This overexpression was not the result of genomic amplification, because the 13q31.3 locus encoding the mir-17-92 cluster was balanced in all the 18 cases assessed by array-CGH (data not shown). We also observed this upregulation in comparison to other vascular tumors and a group of 44 nonvascular sarcomas (well-differentiated/dedifferentiated liposarcomas, WDLPS/DDLPS) without *MYC* amplification/overexpression and previously analyzed with the same method and in the same laboratory (Ugras et al., 2011) (Fig. 3). Moreover, all the five miRNAs encoded by the 17-92 cluster were overexpressed except miR-19b-1 and miR-92a-1, which were poorly represented in AS (data not shown).

**TABLE 2:** Significant Differential Expression of the miR-17-92 Cluster in *MYC*-Amplified AS Compared to *MYC*-unamplified AS, Other Vascular Tumors, WDLPS and DDLPS

	Mean clone count		Frequency of cloning (log scale)			
				
MicroRNA	*MYC*-amplified AS	*MYC*-unamplified AS	MYC-amplified AS	*MYC*-unamplified AS	FC	FDR
hsa-miR-17-92 cluster	36,687	15,609	6.54E−02	1.48E−02	4.4	4E−02
hsa-miR-17	12,999	5,137	2.3E−02	6.7E−03	3.4	1.8E−02
hsa-miR-18a	4,160	1,334	7.7E−03	1.3E−03	5.7	3.3E−02
hsa-miR-19a	10,107	4,744	1.9E−02	5.3E−03	3.6	1.2E−01
hsa-miR-20a	9,357	4,344	1.6E−02	4.2E−03	3.8	3.5E−02
hsa-miR-92a	19	5	3.8E−05	1.3E−05	2.8	4.4E−02

MicroRNA	*MYC*-amplified AS	Other vascular tumors	MYC-amplified AS	Other vascular tumors	FC	FDR
hsa-miR-17-92 cluster	36,687	7,908	6.54E−02	6.89E−03	9.5	4.4E−04
hsa-miR-17	12,999	2,606	2.29E−02	2.32E−03	9.8	1.1E−03
hsa-miR-18a	4,160	609	7.68E−03	5.24E−04	14.6	5.8E−04
hsa-miR-19a	10,107	2,305	1.89E−02	2.01E−03	9.4	2.2E−03
hsa-miR-20a	9,357	2,326	1.59E−02	1.98E−03	8.0	5.1E−03

MicroRNA	*MYC*-amplified AS	WDLPS	MYC-amplified AS	WDLPS	FC	FDR
hsa-miR-17-92 cluster	36,687	2,551	6.54E−02	5.4E−03	12.1	2.2E−07
hsa-miR-17	12,999	277	2.3E−02	4.7E−04	48.4	2.8E−12
hsa-miR-18a	4,160	158	7.7E−03	2.9E−04	26.3	1.8E−08
hsa-miR-19a	10,107	1,374	1.9E−02	3.1E−03	6	7.7E−04
hsa-miR-20a	9,357	742	1.6E−02	1.5^E^−03	10.8	4.8E−06
hsa-miR-92a	19	0	3.8E−05	0	NA	2.9E−10

MicroRNA	*MYC*-amplified AS	DDLPS	MYC-amplified AS	DDLPS	FC	FDR
hsa-miR-17-92 cluster	36,687	2,551	6.54E−02	5.4E−03	12.1	2.2E−07
hsa-miR-17	12,999	549	2.3E−02	5.9E−04	39	2.6E−06
hsa-miR-18a	4,160	274	7.7E−03	3.3E−04	23.1	5.2E−05
hsa-miR-20a	9,357	1,444	1.6E−02	1.7E−03	9.4	1.9E−03

FC: fold-change; FDR: false discovery rate.

### Overexpression of the miR-17-92 Cluster in AS with *MYC* Amplification is Associated with Downregulation of THBS1

**Figure 4:**
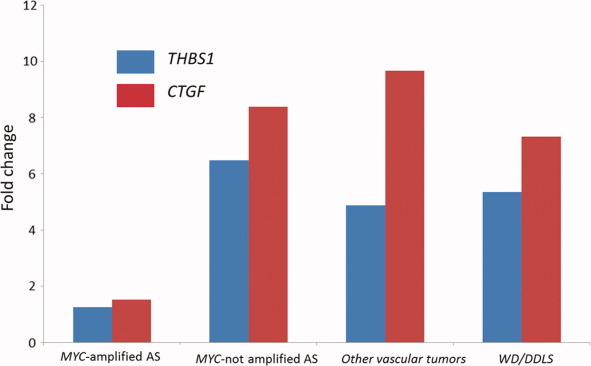
QRT-PCR analysis measuring *THBS1* and *CTGF* mRNA expression in *MYC*-amplified ASs, in MYC-unamplified AS, in other vascular tumors, in WDLPS/DDLPS. Gene expression was quantified by QRT-PCR, and expressed as mean relative expression (reference gene: *GAPDH*).

The miR-17-92 cluster contains miR-18a and miR-19a that have been shown to affect tumor angiogenesis by downregulating the mRNA expression of *THBS1* and CTGF genes, respectively. Therefore, we compared the expression of these two genes in AS with (*n* = 13) and AS without (*n* = 12) *MYC* amplification, other vascular tumors (*n* = 2), and WD/DDLPS (*n* = 4) by qRT-PCR. We found a significant downregulation of *THBS1* and *CTGF* in *MYC*-amplified AS in comparison with *MYC*-unamplified AS (*THBS1*: fold change = 0.15, *P* = 0.02; *CTGF:* fold change = 0.18; *P* = 0.06), other vascular tumors (*THBS1*: fold change = 0.20, *P* = 0.004; *CTGF*: fold change = 0.16; *P* = 0.02) and WD/DDLPS (*THBS1*: fold change = 0.19, *P* = 0.004; *CTGF*: fold change = 0.21; *P* = 0.05) (Fig. 4).

## DISCUSSION

We herein report that genomic amplification of *MYC* is not restricted to radiation-induced AS as previously recognized, but may also occur in a proportion of primary AS. The three cases of primary AS with an amplification of *MYC* arose in different anatomical sites: breast (*n* = 2) and bone (*n* = 1), suggesting that this genomic event represents a true driver genomic event rather than a site-specific epiphenomenon. A recent study which identified a signature of 135 genes discriminating radiation-induced sarcomas (leiomyosarcomas, osteosarcomas, and ASs) from sporadic sarcomas did not include *MYC* as part of this signature; the only function found to be significantly deregulated between the two groups being mitochondria (Hadj-Hamou et al., 2011). Moreover, we reported in a previous study the absence of *MYC* amplification in radiation-induced sarcomas other than ASs (Guo et al., 2011). Altogether, these data suggest that *MYC* amplification is not the hallmark of ionizing radiation in the radiation-induced sarcomas, whatever their histology might be.

By extending the number of primary AS cases analyzed by array-CGH and FISH, we report that genomic amplification of MYC is not restricted to radiation-induced AS as suggested previously, but may also occur in a proportion of primary AS. The high incidence of *MYC* amplification in secondary AS and its occurrence in a subset of primary AS raises the question of its functional role in the tumorigenesis of these highly aggressive vascular tumors. MYC exerts its transcriptional activation function through heterodimerization with MAX (Kretzner et al., 1992). The MYC/MAX heterodimer interacts with specific consensus sequences—the E boxes—in promoters of activated target genes. We previously showed that the MYC/MAX interaction is detected only in AS with *MYC* amplification (Guo et al., 2011). Moreover, we observed that *MAX* mRNA expression did not parallel the high levels of *MYC* in these tumors but showed equally low expression at both mRNA and protein level in AS with and without *MYC* amplification (Guo et al., 2011). These results suggested that the functional role of MYC in *MYC*-amplified AS may involve additional or alternative mechanisms outside the MYC/MAX interaction.

Interestingly, recent studies have demonstrated that besides its involvement in the control of cell proliferation, apoptosis, and differentiation, MYC contributes also in noncell-autonomous cancer process such as angiogenesis (Baudino et al., 2002; Dews et al., 2006; Gordan et al., 2007; Dang et al., 2008). The role of MYC in angiogenesis may be of particular importance in vascular tumors, such as AS. Interestingly, one of the major proangiogenic events triggered by MYC relies on the activation of the miR-17-92 cluster (Dews et al., 2006). This miRNA cluster, located at 13q31.3, encodes six mature miRNAs: miR-17, miR-18a, miR-19a, miR-19b-1, miR-20a, and miR-92a-1 and is a direct transcriptional target of MYC. These miRNAs have been shown to mediate the proangiogenic effect of MYC by decreasing the *THBS1* (particularly for miR-19a) and *CTGF* mRNA (particularly for miR-18a) half-life, thereby promoting tumor growth in vivo in a mouse colon carcinoma model (Dews et al., 2006). THBS1 is the first endogenous inhibitor of angiogenesis which has been identified. This protein inhibits angiogenesis directly by interacting with specific receptors and stimulating Fas/Fas ligand-mediated apoptosis of endothelial cells, but also indirectly by modulating the activity of several angiogenic factors such as FGF-2, VEGF, HGF, or PDGF. There are only very limited data about the status of THBS1 in sarcomas, a decreased expression having been reported in Kaposi sarcoma (Taraboletti et al., 1999) or Ewing sarcoma (Potikyan et al., 2007). Interestingly, the therapeutic efficacy of the THBS1-mimetic angiogenesis inhibitor ABT-510 has been evaluated in a phase 2 trial including several histological sarcoma subtypes (Baker et al., 2008). Although some patients experienced prolonged disease stabilization, the activity of this agent was considered as modest by the investigators. This conclusion may have been affected by the lack of selection of patients included in the study. Indeed, in regard to AS, our results suggest that only a subset of patients may benefit from such a therapeutic strategy.

CTGF (also known as CCN2) is a member of the so-called CCN family, which includes cysteine-rich 61 [(Cyr61) CCN1], nephroblastoma overexpressed [(Nov) CCN3], Wisp-1/elm1 (CCN4), Wisp-2/rCop1 (CCN5), and Wisp-3 (CCN6) cells (Bradham et al., 1991). Its role in angiogenesis appears ambivalent and depending on the cellular context (Inoki et al., 2002). Our results showing a downregulation of CTGF in MYC-amplified AS favor its involvement in angiogenesis inhibition at least in this specific sarcoma subtype. Besides downregulating THBS1, the mir-17-92 cluster can also promote angiogenesis by attenuating the TGF-β signaling pathway to shut down clusterin expression (Dews et al., 2010).

This cluster can also act as a bona fide oncogene. For instance, miR-17 and miR-20a have been shown to regulate cell-cycle progression by targeting E2F1 (O'Donnell et al., 2005; Sylvestre et al., 2007; Woods et al., 2007). However, the analysis of our array-expression data did not reveal any significant difference of expression of E2F1 between MYC-amplified and MYC-unamplified AS (data not shown). This result suggests that noncell-autonomous cancer processes such as angiogenesis represent the main functional consequences of *MYC* amplification in AS.

Array-CGH data confirmed the presence of genomic imbalances in AS cases without *MYC* amplification. However, further studies are necessary to identify the oncogenic trigger events of this subset of tumors. Our CGH results showed coamplification of the 5q35.3 region in 2 out of 11 cases with *MYC* amplification. Our previous study suggested *FLT4* gene as a potential target gene of this amplicon (Guo et al., 2011). Although, our present results support these findings, they identify *MAML1* as a new potential candidate. MAML1 belongs to a family of defined transcriptional coactivators for the Notch pathway (Wu et al., 2000). The Notch pathway plays a crucial role in vascular development and tumor angiogenesis (Ranganathan et al., 2011). There are also several lines of evidence of an aberrant activation of the Notch pathway in benign vascular tumors such as hemangiomas (Calicchio et al., 2009; Wu et al., 2010; Adepoju et al., 2011). As several inhibitors of the Notch pathway are currently under clinical development, the finding of *MAML1* amplification and overexpression in AS deserves further in vitro and in vivo studies to clarify the functional role of the Notch pathway in AS-genesis and as potential therapeutic target.

AS represents a heterogeneous group of malignant vascular tumors, occurring not only in different anatomical locations, but also in distinct clinical settings, such as after radiation therapy or in association with chronic lymphedema. This clinical heterogeneity mirrors the genetic heterogeneity of AS. We have previously shown that despite their consistent morphology, radiation-induced ASs are genetically different from the majority of primary AS as a result of *MYC* amplification. Our present results strongly suggest that this genomic aberration may play a crucial role in the angiogenic phenotype radiation-induced AS and a minority of primary AS through upregulation of the miR-17-92 cluster. Functional experiments are needed to confirm the role of miR-17-92 overexpression and *THBS1* downregulation in *MYC*-amplified AS.
