# Supplementary material for: The miR-17-92 Cluster and Its Target THBS1 Are Differentially Expressed in Angiosarcomas Dependent on MYC Amplification
Source: Genes Chromosomes Cancer. 2012 Mar 2;51(6):569–78. doi: 10.1002/gcc.21943 (PMC3360479; doi:10.1002/gcc.21943)
Supplement: Supplementary file 1 [file gcc0051-0569-SD1.doc]

| **Supplementary Table 1.** Summary of genomic imbalances identified in the 18 AS cases evaluated by array-CGH.* | | | | | | | | | | |
| --- | --- | --- | --- | --- | --- | --- | --- | --- | --- | --- |
|  | | **All aberrations** | | | **All gains** | | | **All losses** | | |
| **Case** | **Subtype** | **Total nr of aberrations** | **Total interval spanned (bp)** | **Mean size (bp)** | **Total nr of aberrations** | **Total interval spanned (bp)** | **Mean size (bp)** | **Total nr of aberrations** | **Total interval spanned (bp)** | **Mean size (bp)** |
| AS3 | primary | 35 | 328607509 | 9388786 | 21 | 292551019 | 13931001 | 14 | 36056490 | 2575464 |
| AS9 | primary | 19 | 337061448 | 17740076 | 4 | 135288163 | 33822041 | 15 | 201773285 | 13451552 |
| AS11 | primary | 20 | 16601136 | 830057 | 10 | 5473983 | 547398 | 10 | 11127153 | 1112715 |
| AS70 | primary | 10 | 22672442 | 2267244 | 6 | 7546283 | 1257714 | 4 | 15126159 | 3781540 |
| AS124 | primary | 35 | 521149084 | 14889974 | 14 | 321930089 | 22995006 | 21 | 199218995 | 9486619 |
| AS125 | primary | 10 | 4549128 | 454913 | 5 | 3274639 | 654928 | 5 | 1274489 | 254898 |
| AS10 | secondary | 22 | 389670377 | 17712290 | 13 | 382158161 | 29396782 | 9 | 7512216 | 834691 |
| AS15 | secondary | 13 | 18118247 | 1393711 | 5 | 3413660 | 682732 | 8 | 14704587 | 1838073 |
| AS20 | secondary | 13 | 10698268 | 822944 | 7 | 6254915 | 893559 | 6 | 4443353 | 740559 |
| AS29 | secondary | 12 | 14461073 | 1205089 | 3 | 299281 | 99760 | 9 | 14161792 | 1573532 |
| AS30 | secondary | 38 | 923405420 | 24300143 | 12 | 255688813 | 21307401 | 26 | 667716607 | 25681408 |
| AS32 | secondary | 24 | 202266196 | 8427758 | 5 | 4194599 | 838920 | 19 | 198071597 | 10424821 |
| AS38 | secondary | 22 | 114531845 | 5205993 | 10 | 94842361 | 9484236 | 12 | 19689484 | 1640790 |
| AS39 | secondary | 24 | 1140781390 | 47532558 | 9 | 299343008 | 33260334 | 15 | 841438382 | 56095892 |
| AS68 | secondary | 21 | 422063597 | 20098267 | 8 | 384012479 | 48001560 | 13 | 38051118 | 2927009 |
| AS73 | secondary | 49 | 1825169133 | 37248350 | 31 | 1553693061 | 50119131 | 18 | 270949666 | 15052759 |
| AS76 | secondary | 28 | 525899131 | 18782112 | 10 | 140106399 | 14010640 | 18 | 385792732 | 21432930 |
| AS123 | secondary | 43 | 355162223 | 8259587 | 21 | 86086232 | 4099344 | 22 | 269075991 | 12230727 |

*****For each case the total number of discrete aberrations identified is defined, and then categorized to indicate the relative proportion of genomic gains and losses contributing to this total. The physical size of the genome spanned by copy number aberrations is also indicated for all three categories (all aberrations, all gains and all losses). Based on these data a mean aberration size is calculated for each case.
